# Lower carbohydrate and higher fat intakes are associated with higher hemoglobin A1c: findings from the UK National Diet and Nutrition Survey 2008–2016

**DOI:** 10.1007/s00394-019-02122-1

**Published:** 2019-11-04

**Authors:** Chaitong Churuangsuk, Michael E.J. Lean, Emilie Combet

**Affiliations:** Human Nutrition, School of Medicine, Dentistry and Nursing, College of Medical, Veterinary and Life Sciences, University of Glasgow, New Lister Building, Glasgow Royal Infirmary, Room 2.22, Level 2, 10-16 Alexandra Parade, Glasgow, G31 2ER UK

**Keywords:** Reduced carbohydrate diet, Carbohydrate-restricted diet, High-fat diet, Dietary recommendation, Glycated hemoglobin, Type 2 diabetes

## Abstract

**Purpose:**

Evidence of low-carbohydrate, high-fat diets (LCHF) for type 2 diabetes (T2DM) prevention is scarce. We investigated how carbohydrate intake relates to HbA1c and T2DM prevalence in a nationally representative survey dataset.

**Methods:**

We analyzed dietary information (4-day food diaries) from 3234 individuals aged ≥ 16 years, in eight waves of the UK National Diet and Nutrition Survey (2008–2016). We calculated LCHF scores (0–20, higher score indicating lower  %food energy from carbohydrate, with reciprocal higher contribution from fat) and UK Dietary Reference Value (DRV) scores (0–16, based on UK dietary recommendations). Associations between macronutrients and diet scores and diabetes prevalence were analyzed (in the whole sample) using multivariate logistic regression. Among those without diabetes, analyses between exposures and %HbA1c (continuous) were analyzed using multivariate linear regression. All analyses were adjusted for age, sex, body mass index, ethnicity, smoking status, total energy intake, socioeconomic status and survey years.

**Results:**

In the overall study sample, 194 (6.0%) had diabetes. Mean intake was 48.0%E for carbohydrates, and 34.9%E for total fat. Every 5%E decrease in carbohydrate, and every 5%E increase in fat, was associated with 12% (95% CI 0.78–0.99; *P *= *0.03*) and 17% (95% CI 1.02–1.33; *P *= 0.02) higher odds of diabetes, respectively. Each two-point increase in LCHF score is related to 8% (95% CI 1.02–1.14; *P *= 0.006) higher odds of diabetes, while there was no evidence for association between DRV score and diabetes. Among the participants without diagnosed diabetes (*n* = 3130), every 5%E decrease in carbohydrate was associated with higher %HbA1c by + 0.016% (95% CI 0.004–0.029; *P *= 0.012), whereas every 5%E increase in fat was associated with higher  %HbA1c by + 0.029% (95% CI 0.015–0.043; *P *< 0.001). Each two-point increase in LCHF score is related to higher  %HbA1c by + 0.010% (0.1 mmol/mol), while each two-point increase in the DRV score is related to lower  %HbA1c by − 0.023% (0.23 mmol/mol).

**Conclusions:**

Lower carbohydrate and higher fat intakes were associated with higher HbA1c and greater odds of having diabetes. These data do not support low(er) carbohydrate diets for diabetes prevention.

**Electronic supplementary material:**

The online version of this article (10.1007/s00394-019-02122-1) contains supplementary material, which is available to authorized users.

## Introduction

Diabetes prevalence is predicted to rise from 425 million people in 2017 to 629 million people by 2045 [1]. It is clear that body fat accumulation is the dominant factor behind development of type 2 diabetes mellitus (T2DM), and consistent evidence shows that reversing that process by weight loss is the key mechanism for prevention, as well as for remission of established T2DM [2]. Several large diabetes prevention trials have shown modest weight loss to be effective, principally by lowering fat intake (< 30%E) along with lifestyle modification [3–6].

It has been hypothesized that lower postprandial glucose excursion on a low-carbohydrate, high-fat (LCHF) diet may lead to a better glucose control [7, 8]. LCHF diets have been promoted as a possible strategy for the management and prevention of T2DM, but there is no consensus on the best macronutrient composition [9]. LCHF diets can certainly lead to weight loss and reduce HbA1c, but there is no clear evidence of LCHF diet superiority over other dietary approaches, with most of meta-analyses of low to moderate methodological quality [10–13]. Furthermore, there are indications that carbohydrate-restricted diets may not provide the full complement of micronutrients leading to potential insufficiencies in vitamins and minerals [14].

Longitudinal cohort studies have not shown conclusive associations between LCHF diets and long-term risk of T2DM [15–17]. No LCHF diet trial (whether or not as part of a wider lifestyle modification) has yet been conducted aiming to prevent incident T2DM [18].

The present study was conducted using the National Diet and Nutrition Survey (NDNS), the largest UK database of a continuous, cross-sectional survey for nutrition and health, using prospective dietary records and providing information on biomarkers. It investigated the relationships of reported dietary carbohydrate, fat, saturated fat, LCHF dietary patterns and adherence to dietary recommendations with HbA1c concentration (as a marker of diabetes and heart disease risks) and with risk of T2DM.

## Subjects and methods

### Study design

The present study is a secondary analysis of publicly available data from the NDNS rolling program 2008–2016. This is a continuous cross-sectional survey providing high quality, nationally representative data on food consumption, lifestyle, and health information including laboratory data of micronutrients and metabolic risk factors. Representative samples of individuals aged 1.5 year and over were studied each year, providing a total of 12,097 respondents. Ethical approval for the NDNS was granted by appropriate Local Research Ethics Committees and the data can be accessed online through the UK Data Service [19].

### Population

**Fig. 1:**
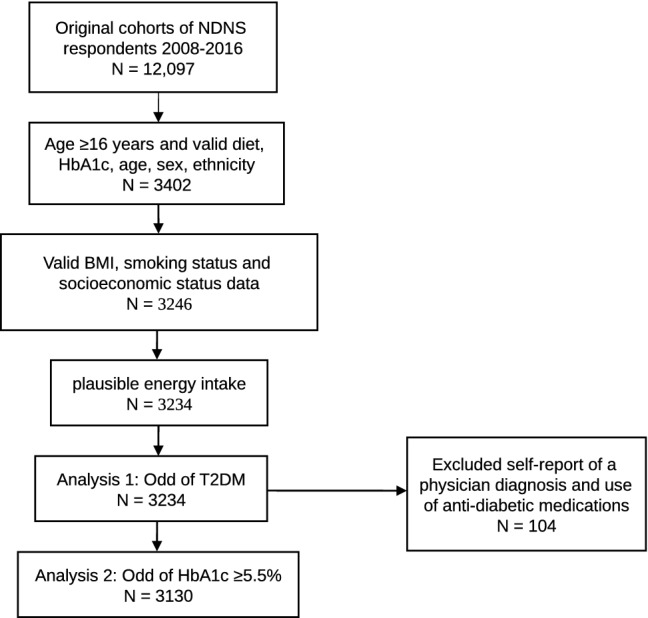
Flowchart of participant inclusion. *NDNS* national diet and nutrition survey, *BMI* body mass index, *T2DM* type 2 diabetes mellitus

Participants were randomly selected from a sample of private households in the UK Postcode Address File. They were included in the present analysis if they were aged ≥ 16 years (the minimum age that could consent to the blood test), had complete information on dietary data, health habits, anthropometric data, blood analysis and had plausible reported energy intake (as an average of 3–4 food diary days) defined as > 600 kcal &  < 4200 kcal per day for men and > 500 kcal &  < 3600 kcal per day for women [20]. The participant inclusion flow chart is shown in Fig. 1.

### Dietary measure and exposure variables

The NDNS dietary assessment is a prospective 4-day food diary (covering 2 weekdays and 2 weekend days) with instructions on how to estimate portion size. This 4-day food diary has been validated against four non-consecutive days interviewer-administered 24-hour recalls, NDNS appendix A [21]. In the present study, exposures were the percentages of energy intake from carbohydrate, protein, fat including saturated fat, and the adherences to LCHF dietary pattern and UK dietary recommendations—all as continuous variables.

Adherence to an LCHF dietary pattern was defined by an ‘LCHF score’ calculated using the percentage of food energy derived from carbohydrate and fat, based on the approach of Sjogren et al. and de Koning et al. [15, 22]. Both carbohydrate and fat intakes were divided into 11 equal groups, assigned a score of 0 to 10. The lowest carbohydrate intake group (≤ 39.5%E) was assigned a score of 10, and the highest carbohydrate intake group was assigned a score of 0. Scoring for fat intake was reversed, the lowest fat intake group was assigned a score of 0, and the highest fat intake group (> 42.9%E) was assigned a score of 10. The overall LCHF score ranged from 0 to 20, with a higher score indicating higher adherence to LCHF diets, lower intake of carbohydrate and higher intake of fat (Online Resource Table 1).

**Table 1:** Population characteristics

	All	Diagnosis of diabetes	Not known to have diabetes (*n* = 3130)				
			HbA1c < 5.5%	HbA1c ≥ 5.5%	HbA1c ≥ 6.5%	Not dieting	Dieting
	*n* = 3234	*n* = 104	*n* = 1613	*n = *1427	*n* = 90	*n* = 2741	*n* = 389
%HbA1c	5.6 ± 0.7	7.7 ± 1.7	5.2 ± 0.2	5.8 ± 0.2	7.4 ± 1.2	5.5 ± 0.5	5.5 ± 0.5
HbA1c ≥ 5.5% (%)	–	–	–	–	–	1323 (48.3)	194 (49.9)
Dieting (%)	441 (12.7)	22 (21.2)	195 (12.1)	180 (12.6)	14 (15.6)	–	–
Age, years	46.3 ± 18.5	58.6 ± 14.4	37.9 ± 16.1	53.9 ± 17.1	62.8 ± 15.2	45.9 ± 18.9	45.9 ± 15.5
Sex: male (%)	1379 (42.6)	67 (64.4)	663 (41.1)	607 (42.5)	42 (46.7)	1207 (44.0)	105 (27.0)
BMI (kg/m^2^)	27.1 ± 5.4	31.8 ± 6.4	25.9 ± 5.0	27.9 ± 5.3	31.6 ± 5.7	26.5 ± 5.1	30.5 ± 5.2
Ethnicity: White (%)	3025 (93.5)	92 (88.5)	1521 (94.3)	1330 (93.2)	82 (91.1)	2568 (93.7)	365 (93.8)
Smoking (%)							
Current smoker	664 (20.5)	12 (11.5)	314 (19.5)	323 (22.6)	15 (16.7)	603 (22.0)	49 (12.6)
Ex-smoker	718 (22.2)	37 (35.6)	304 (18.8)	345 (24.2)	32 (35.6)	574 (20.9)	107 (27.5)
Never	1852 (57.3)	55 (52.9)	995 (61.7)	759 (53.2)	43 (47.8)	1564 (57.1)	233 (59.9)
Socioeconomic status (%)							
Higher professional	497 (15.4)	17 (16.3)	283 (17.5)	188 (13.2)	9 (10)	433 (15.8)	47 (12.1)
Lower professional	866 (26.8)	20 (19.2)	447 (27.7)	387 (27.1)	12 (13.3)	740 (27.0)	106 (27.2)
Intermediate occupations	316 (9.8)	6 (5.8)	168 (10.4)	133 (9.3)	9 (10)	265 (9.7)	45 (11.6)
Small employers	339 (10.5)	13 (12.5)	143 (8.9)	170 (11.9)	13 (14.4)	280 (10.2)	46 (11.8)
Technical occupations	312 (9.6)	15 (14.4)	155 (9.6)	133 (9.3)	9 (10)	254 (9.3)	43 (11.1)
Semi-routine occupations	429 (13.3)	15 (14.4)	205 (12.7)	196 (13.7)	13 (14.4)	363 (13.2)	51 (13.1)
Routine occupations	357 (11.0)	18 (17.3)	141 (8.7)	180 (12.6)	18 (20)	298 (10.9)	41 (10.5)
Never worked/Other	118 (3.6)	–	71 (4.4)	40 (2.8)	7 (7.8)	108 (3.9)	10 (2.6)
Total Energy (kcal)	1815.4 ± 551.4	1770.7 ± 575.2	1844.9 ± 556.5	1795.0 ± 543.1	1660.7 ± 523.9	1845.4 ± 555	1612.9 ± 472
Carbohydrate (g/day)	221.4 ± 71.0	210.7 ± 69.6	226.5 ± 73.1	217.7 ± 68.4	202.0 ± 66.9	225.3 ± 71.6	197.2 ± 62.1
Carbohydrate (%E)	48.0 ± 6.6	46.6 ± 7.0	48.6 ± 6.6	47.6 ± 6.5	47.1 ± 6.7	48.1 ± 6.5	47.8 ± 7.1
Fat (g/day)	68.2 ± 25.8	68.6 ± 27.4	68.2 ± 25.7	68.3 ± 25.8	64.0 ± 24.4	69.5 ± 25.8	58.4 ± 22.8
Fat (%E)	34.9 ± 6.2	35.5 ± 6.2	34.5 ± 6.3	35.3 ± 6.0	35.4 ± 6.3	35.1 ± 6.0	33.4 ± 7.0
Saturated fat (g/day)	25.4 ± 11.0	25.6 ± 11.4	25.1 ± 10.8	25.8 ± 11.1	24.5 ± 11.5	26.0 ± 11.0	21.5 ± 9.8
Saturated fat (%E)	13.0 ± 3.3	13.1 ± 3.4	12.6 ± 3.3	13.3 ± 3.4	13.3 ± 3.6	13.1 ± 3.3	12.2 ± 3.5
Protein (g/day)	72.6 ± 22.6	75.2 ± 24.5	72.7 ± 23.1	72.5 ± 22.0	69.0 ± 21.4	72.6 ± 22.6	71.5 ± 22.1
Protein (%E)	17.1 ± 3.8	18.0 ± 4.3	16.9 ± 3.9	17.2 ± 3.6	17.5 ± 3.9	16.8 ± 3.6	18.8 ± 4.2
Non-starch polysaccharide (g/day)	13.7 ± 5.0	14.1 ± 5.5	13.7 ± 5.0	13.7 ± 4.9	13.1 ± 5.2	13.7 ± 5.0	13.6 ± 4.8
Fruit and Vegetables (g/day)	277.4 ± 172.1	273.5 ± 181.9	271.8 ± 172.9	283.5 ± 169.1	283.7 ± 192.1	271.9 ± 166.2	316.6 ± 203.3
LCHF score, median (IQR)	10 (5, 15)	12 (6, 17)	10 (4, 15)	10 (6, 15)	11 (6, 16)	10 (5, 15)	9 (4, 15)
DRV score, median (IQR)	6 (4, 8)	5 (4, 8)	5 (4, 8)	6 (4, 8)	5 (4, 9)	5 (4, 8)	6 (4, 9)

Data are mean ± SD unless otherwise indicated

Adherence to UK dietary recommendations (Dietary Reference Value, ‘DRV score’) was calculated according to the method by Eriksen and colleagues, derived from 8 nutrients and foods: carbohydrate  %food energy, fat  %food energy, saturated fat  %food energy, sugar (as non-milk extrinsic sugar, NMES)  % food energy, fruit and vegetables in grams of intake, fish in grams of intake, sodium in mg of intake, and fiber (as non-starch polysaccharide, NSP) in grams of intake [23]. DRV score ranged from 0 to 16, with higher DRV scores indicative of a higher adherence to UK dietary recommendations (Online Resource Table 2).

**Table 2:** Associations between %HbA1c concentration and macronutrients and dietary adherence scores in subjects without diagnosed diabetes (*n* = 3130)

Predictors	*β*^a^	SE	Lower 95% CI	Upper 95% CI	*P* value
Macronutrients^b^					
Carbohydrate	− 0.016	0.007	− 0.029	− 0.004	0.012
Fat	0.029	0.007	0.015	0.043	< 0.001
Saturated fat	0.051	0.013	0.025	0.078	< 0.001
Protein	− 0.027	0.012	− 0.050	− 0.004	0.024
Adherence score^c^					
LCHF score	0.010	0.003	0.004	0.016	0.001
DRV score	− 0.023	0.006	− 0.035	− 0.012	< 0.001

*LCHF* low carbohydrate high fat, *DRV* dietary reference values, *CI* confidence interval, *SE* standard error

^a^Multivariate linear regression model with regression coefficients (*β*) in %HbA1c change for 5% food energy increment and 2-point diet score increment adjusted for age, sex, BMI, ethnicity, smoking status, socioeconomic status, survey year, and energy intake

^b^Per 5% food energy increment

^c^Per 2-point score increment

### Covariates

The following variables were determined a priori as potentially affecting HbA1c and/or diet composition: age, sex, ethnicity (White, Asian, Black, Mixed, and other), body mass index (BMI), smoking status (current smoker, ex-smoker, never smoked), energy intake, socioeconomic status defined by the National Statistics Socio-Economic Classification (NS-SEC), and survey year. All covariate information including whether currently on a weight-loss diet was gathered during face-to-face interviews conducted by the NDNS researchers. Body weight was measured by a scale and body height was measured by a stadiometer during the interview. BMI is calculated by body weight (kg) divided by a square of height (m).

### Outcome variables

Two primary outcomes were defined. The primary outcome 1, having diabetes, was analyzed using the whole dataset. Diabetes status was either diagnosed diabetes (based on self-report of a physician diagnosis and/or use of anti-diabetic medications) or HbA1c ≥ 6.5% in those who had never been diagnosed before. The NDNS dataset does not break down type 1 and type 2 diabetes, nor insulin use. Therefore, we conducted a sensitivity analysis by excluding possible T1DM from the dataset (further details in the statistical analysis section below).

The primary outcome 2 was HbA1c concentration among those without diabetes. Therefore, participants with diagnosed diabetes (based on self-report of a physician diagnosis and/or use of anti-diabetic medications) were excluded to reduce bias from reverse causality, as diagnosis and treatment would be likely to affect both diets and HbA1c (e.g., those with diagnosed diabetes might have reduced carbohydrate intake). HbA1c was analyzed as a continuous variable to investigate how carbohydrates and diets relate to HbA1c concentration. We also investigated how dietary carbohydrate relates to cardiovascular disease risk, by categorizing HbA1c into a binary outcome, using a cut point of ≥ 5.5% as a clinically meaningful threshold (2.5–7.8% per 100 person-year increase in T2DM annual incident rate, 21–61% increase in cardiovascular death; compared to HbA1c <5.5%) [24, 25].

### Statistical analysis

Only participants who had complete data for all variables were included in the analysis. Men and women were combined in the analysis to increase the power, as there was no difference in diet recommendation between sexes. Data are presented as percentage of total, means and standard deviations (unless otherwise stated).

*Primary outcome 1:* associations between T2DM status and dietary exposures (macronutrients as percentage of food energy and diet scores) were analyzed using multivariate logistic regression with adjustment for covariates. Analyses were conducted in the whole dataset. Because the dataset does not allow T1DM and T2DM to be identified separately, we carried out two sensitivity analyses. There is a greater likelihood of T1DM among people with diabetes at younger ages. We firstly conducted a sensitivity analysis by excluding all participants age under 30 years.

We carried out another sensitivity analysis by randomly excluding 10% of participants with diabetes, based on the overall T1DM prevalence of approximately 10% of total diabetes prevalence, weighted by age groups [26]. In the main NDNS dataset, there are 104 participants with diabetes, which means that about 10 may have T1DM. We, therefore, did not just randomly exclude 10% of participants with diabetes. Instead, this analysis was based on 1000 separate analyses, with random sampling and exclusions of 10 different participants with diabetes, weighted for age (online Resource Table 3 shows the prevalence of T1DM in all patients with diabetes, stratified by age groups). The recent National Diabetes Audit Report showed that T1DM presents in all age groups with higher proportion in younger age (67.4% in those aged 20–29 years with diabetes) compared to older age (3.4% in those aged 60–69 years with diabetes). We selected the 10% for exclusion using the number reflecting the prevalence of T1DM in each age group [26]. This sensitivity analysis aimed to explore how much estimates (Odds Ratio) differed when excluding possible T1DM patients from the analyses.

**Table 3:** Sensitivity analyses in subjects without diagnosed diabetes aged ≥ 18 years (*n* = 2865), and not being on a weight-loss diet (*n* = 2741) showing associations between %HbA1c and macronutrients and diet scores

	HbA1c ≥ 5.5%			%HbA1c concentration				
Predictors	Odds Ratio^a^	95% CI	*P* value	*β*^b^	SE	Lower 95% CI	Upper 95% CI	*P* value
Age ≥ 18 years (*n* = 2865)								
Macronutrients^c^								
Carbohydrate	0.94	0.88–1.00	0.046	− 0.015	0.007	− 0.028	− 0.002	0.020
Fat	1.14	1.06–1.22	< 0.001	0.027	0.007	0.013	0.041	< 0.001
Saturated fat	1.26	1.10–1.44	< 0.001	0.044	0.013	0.018	0.070	< 0.001
Protein	0.88	0.78–0.99	0.034	− 0.025	0.012	− 0.048	− 0.001	0.039
Adherence score^d^								
LCHF score	1.04	1.01–1.07	0.014	0.009	0.003	0.004	0.015	0.001
DRV score	0.91	0.86–0.97	0.002	− 0.020	0.006	− 0.031	− 0.008	< 0.001
No weight-loss diet (*n* = 2741)								
Macronutrients^c^								
Carbohydrate	0.94	0.88–1.01	0.085	− 0.014	0.007	− 0.028	0.000	0.051
Fat	1.11	1.03–1.20	0.005	0.026	0.008	0.011	0.042	0.001
Saturated fat	1.24	1.08–1.42	0.003	0.050	0.014	0.022	0.079	0.001
Protein	0.91	0.80–1.03	0.145	− 0.026	0.013	− 0.052	0.000	0.048
Adherence score^d^								
LCHF score	1.03	1.00–1.06	0.046	0.009	0.003	0.003	0.015	0.004
DRV score	0.91	0.85–0.96	0.001	− 0.024	0.006	− 0.036	− 0.011	< 0.001

*LCHF* low carbohydrate high fat, *DRV* dietary reference values, *CI* confidence interval, *SE* standard error

^a^Multivariate logistic regression adjusted for age, sex, BMI, ethnicity, smoking status, socioeconomic status, survey years, total energy intake

^b^Multivariate linear regression adjusted for age, sex, BMI, ethnicity, smoking status, socioeconomic status, survey years, total energy intake

^c^Per 5% food energy increment

^d^Per 2-point score increment

We also conducted a third sensitivity analysis by excluding individuals with known (diagnosed) diabetes (*n* = 104 out of 194) to explore the associations between diets and unknown T2DM prevalence (*n* = 90). Finally, a fourth sensitivity analysis was conducted by excluding individuals with T2DM being on a weight-loss diet (*n* = 36 out of 194) to explore how much odds ratio differed when excluding the possible effect of weight loss.

*Primary outcome 2*: in participants without diagnosed diabetes, multiple linear regression was conducted to analyze relationships between macronutrients, diet scores and HbA1c concentration, and multivariate logistic regression was used to test associations between diets and having HbA1c ≥ 5.5%. All analyses were adjusted for covariates. Sensitivity analysis was carried out by repeated analysis in (i) people with non-diagnosed diabetes who reported not being on a weight-loss diet; (ii) people with non-diagnosed diabetes aged ≥ 18 years old; and (iii) in subgroup analysis of people with BMI < 25 kg/m^2^ and BMI ≥ 25 kg/m^2^ (with versus without overweight & obesity).

All statistical tests were two sided with an alpha of 0.05. R version 3.5.1 was used with package ‘epical’, ‘rms’ and ‘ggplot2’ for statistical analysis.

## Results

Table 1 shows population characteristics. Between 2008 and 2016, a total of 3234 participants (mean age 46.3 ± 18.5 years, 42.6% men) had complete data and were included in the analysis. Of these, 104 participants (3.2%) had a known diagnosis of diabetes. Among those without a diabetes diagnosis (*n* = 3130), mean HbA1c was 5.5 ± 0.5%, *n* = 90 (2.8%) had HbA1c ≥ 6.5%, *n* = 365 (11.7%) had HbA1c ≥ 6.0%, and *n* = 1517 (48.5%) had an HbA1c ≥ 5.5%. Participants with either diabetes or HbA1c ≥ 5.5% were older (59 ± 14 and 54 ± 17 years, respectively) and had a higher BMI (32 ± 6 and 28 ± 5 kg/m^2^, respectively) than those with lower HbA1c (age 38 ± 16 years, BMI 26 ± 5 kg/m^2^). Most participants were white (93.5%) and non-smokers (57.3%), and 12% were following self-reported weight-loss diets (Table 1).

Dietary intakes were comparable across groups of diagnosed and non-diagnosed diabetes (Table 1). Mean total energy intake was 1815 ± 551 kcal. Carbohydrate, fat, saturated fat and protein intakes were approximately 48%, 35%, 13% and 17% food energy across all groups. Non-starch polysaccharides intake was low at 13.7 ± 5 g/day (target 18 g/day, Englyst method). Participants who reported being on a weight-loss diet had higher fruits and vegetables intakes (317 ± 203 g/day) than the overall sample population (277 ± 172 g/day), with no difference in LCHF or DRV scores. The distributions of the LCHF and DRV scores are shown in Online Resource Fig. 1. There was a negative correlation between LCHF and DRV scores (rho = − 0.63, *P *< 0.001).

### Primary outcome 1: odds of Type 2 diabetes—analyses in the whole sample

**Fig. 2:**
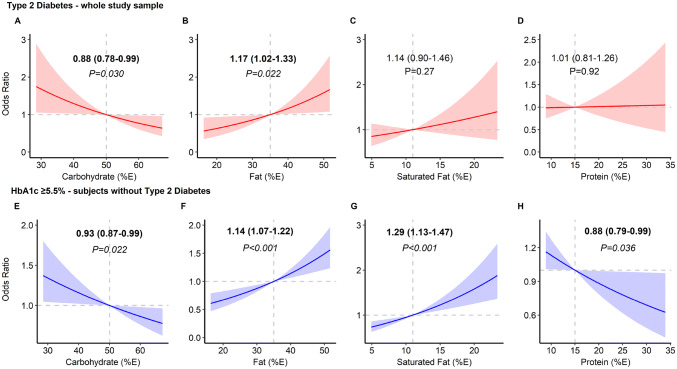
Associations between macronutrients and odds of diabetes (**a**–**d**) and elevated HbA1c ≥ 5.5% (**e**–**f**). Values are odds ratio with 95% confidence interval calculated from multiple variable logistic regression adjusted for age, sex, body mass index, ethnicity, smoking status, socioeconomic status, survey year and total energy intake. Reference levels for regression analysis: carbohydrate, fat, saturated fat and protein are 50%, 35%, 11% and 15% food energy

*Macronutrients*: Out of 3234 participants, 194 participants (6%) were considered as having diabetes. In multivariate logistic regression models adjusted for covariates, an increase of 5% food energy from carbohydrate was associated with a 12% ‘lower odd’ of having diabetes (95% CI 0.78–0.99; *P *= 0.03), while every 5% food energy increase from fat intake was associated with a 17% ‘higher odd’ of diabetes, (95% CI 1.02–1.33; *P *= 0.022). There was no evidence to support the associations between diabetes and saturated fat and protein intakes (Fig. 2a–d).

**Fig. 3:**
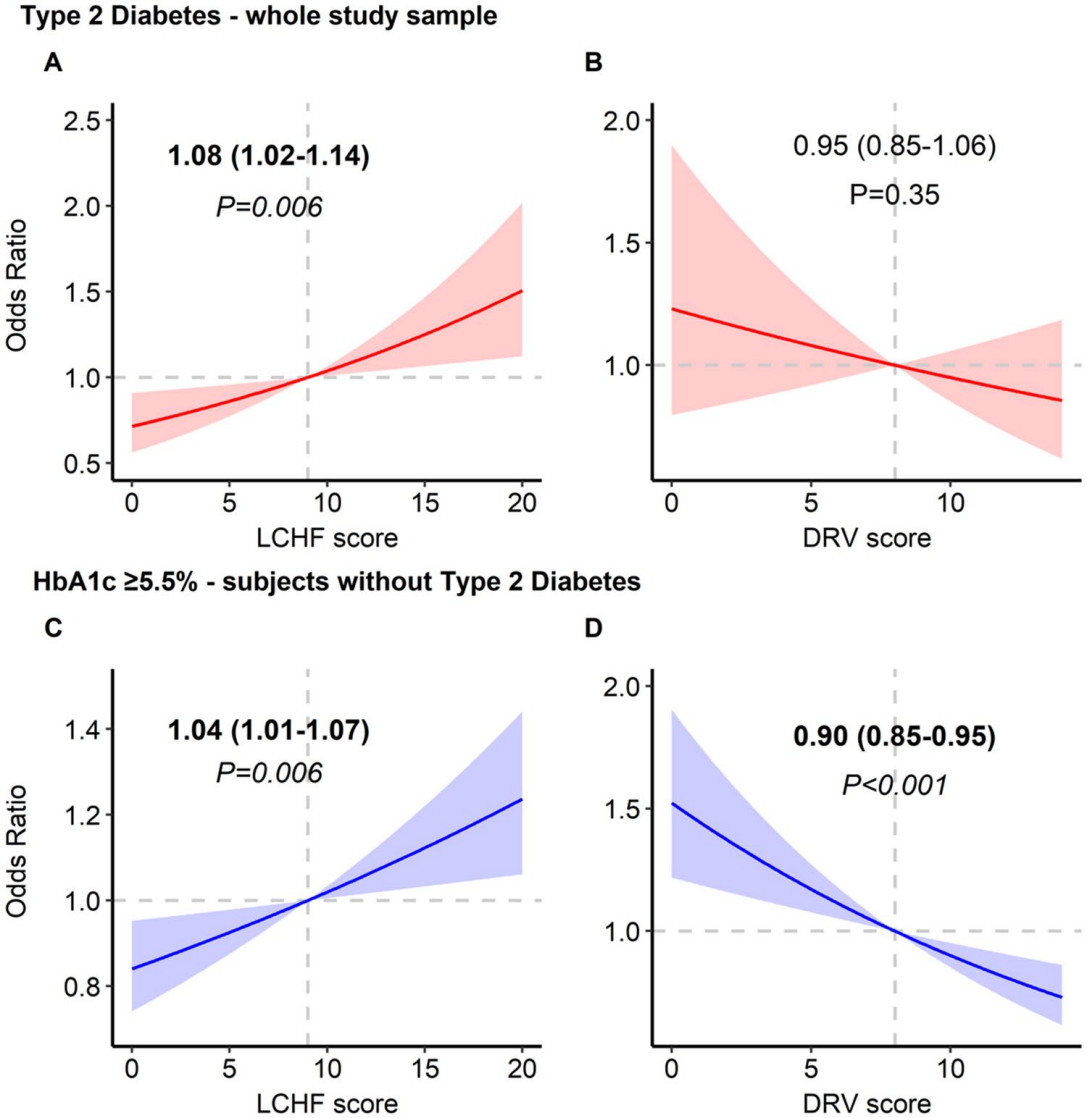
Associations between diet scores and odds of diabetes (**a**, **b**) and elevated HbA1c ≥ 5.5% (**c**, **d**). Values are odds ratio with 95% confidence interval calculated from multiple variable logistic regression adjusted for age, sex, body mass index, ethnicity, smoking status, socioeconomic status, survey year and total energy intake. Reference level for regression analysis is nine for low-carb, high*-*fat (LCHF) score and eight for dietary reference value (DRV) score as these scores reflected intakes that met UK recommendations

*Dietary adherence:* Every 2-point increase in LCHF score was associated with an 8% ‘higher odds’ of diabetes (95% CI 1.02–1.14; *P *= 0.006). However, the evidence did not support an association between diabetes and adherence to the UK dietary recommendations (adjusted OR 0.95; 95% CI 0.85–1.06, *P *= 0.35 for every 2-point increase in DRV score) (Fig. 3a–b).

*Sensitivity analysis:* To investigate how estimates differed when we excluded participants with possible T1DM, we conducted two sensitivity analyses (because the dataset does not breakdown T1DM and T2DM): (1) we excluded all participants with diabetes aged under 30; and (2) we conducted 1000 logistic regression analyses after randomly excluding 10% of population with diabetes (10% prevalence of T1DM in total diabetes prevalence) stratified by age groups. The results of the sensitivity analyses remained similar to the main analyses for each macronutrient and dietary adherence score (Online Resource Table 4). We conclude that there was no major effect on our conclusions from the small number (approximately 10) of T1DM patients who may possibly have been included in the sample. Further sensitivity analyses by excluding individuals with known diabetes (*n* = 104 out of 194) and individuals with diabetes being on weight-loss diet (*n* = 36 out of 194) also showed that the direction of associations remained similar and the magnitude of effect size did not change markedly from the main analyses (Online Resource Table 4).

### Primary outcome 2: HbA1c concentration—analyses in individuals without diagnosed diabetes

We conducted analyses in participants without a diabetes diagnosis (*n* = 3130), to minimize bias from reverse causality as diagnosis and treatment would be likely to affect both diets and HbA1c.

*Macronutrients*: A higher carbohydrate intake (every extra 5%food energy) was associated with lower  %HbA1c (− 0.016% or − 0.16 mmol/mol; 95% CI − 0.029%, − 0.004%; *P *= 0.012). A similar association for protein was found, with every extra 5% food energy from protein associated with lower  %HbA1c (− 0.027% or − 0.27 mmol/mol; 95% CI − 0.050%, − 0.004%; *P *= 0.024). In contrast, fat and saturated fat intakes were both associated with higher  %HbA1c, by + 0.029% (or + 0.29 mmol/mol; 95% CI 0.015%, 0.043%) and + 0.051% (or + 0.51 mmol/mol; 95% CI 0.025%, 0.078%), respectively, *P *< 0.001 (Table 2).

*Dietary adherence:* A higher LCHF score (per two-point increase) was also associated with higher  %HbA1c concentration by + 0.010% (or 0.1 mmol/mol; 95% CI 0.004%, 0.016%; *P *= 0.001), while higher DRV score (per two-point increase) was inversely associated with  %HbA1c by − 0.023% (or 0.23 mmol/mol; 95% CI − 0.035%, − 0.012%; *P *< 0.001) (Table 2).

*Sensitivity analysis:* Similar associations between macronutrients, diet scores and HbA1c concentration remained in the sensitivity analyses with (1) participants aged ≥ 18 years old; (2) participants who were not on a weight-loss diet; and (3) in a subgroup of subjects with BMI ≥25 kg/m^2^ (Table 3 and Online Resource Table 5). However, none of the associations remained significant in individuals with BMI < 25 kg/m^2^, except for saturated fat intake which was associated with higher  %HbA1c (+ 0.04%; 95% CI 0.01%, 0.07%; *P *= 0.021), per 5% food energy increment (Online Resource Table 5).

### Primary outcome 2: Odds of elevated HbA1c (≥ 5.5%), as a cardiovascular disease risk biomarker—analyses in individuals without diagnosed diabetes

*Macronutrients:* Every 5% extra food energy from carbohydrate and protein were associated with 7% (95% CI 0.87–0.99; *P *= 0.022) and 12% (95% CI 0.79–0.99; *P *= 0.036) ‘lower odds’ of elevated HbA1c, respectively. In contrast, every extra 5% food energy from fat and saturated fat were associated with ‘higher odds’ of elevated HbA1c, by 14% (95% CI 1.07–1.22; *P *< 0.001) and 29% (95% CI 1.13–1.47; *P *< 0.001), respectively (Fig. 2e–h).

*Dietary adherence:* associations between diet scores and elevated HbA1c were found. Every 2-point increase in LCHF score was associated with a 4% higher odd of having an elevated HbA1c (95% CI 1.01–1.07; *P *= 0.006), while every 2-point increase in DRV score was *inversely* associated with the odd of elevated HbA1c by 10%, 95% CI 0.85–0.95, *P *< 0.001 (Fig. 3c–d).

*Sensitivity analysis:* A sensitivity analysis in participants aged ≥ 18 years old showed associations consistent with the main analysis (Table 3). Similarly, in participants who were not on weight-loss diet, fat and saturated fat intakes remained strongly associated with 11% (95% CI 1.03–1.20; *P *= 0.005) and 24% (95% CI 1.08–1.42; *P *= 0.003) higher odds of elevated HbA1c (Table 3). Consistently, LCHF score was associated with higher odds of elevated HbA1c, whereas DRV score was associated with lower odds of elevated HbA1c (Table 3). Further sensitivity analysis in subgroup with overweight/obesity (BMI ≥ 25 kg/m^2^) showed similar associations between macronutrients, diet scores and odds of elevated HbA1c. In contrast, no evidence was found for these associations in a subgroup with BMI < 25 kg/m^2^ (Online Resource Table 5 and Online Resource Fig. 2).

## Discussion

There has been very limited evidence supporting the use of LCHF diets for the prevention of T2DM. To our knowledge, this is the first study investigating whether carbohydrate intake and LCHF dietary pattern relate to HbA1c concentration in a population-based cross-sectional study of nutrition and health. Surprisingly, given the enthusiasm for LCHF diets in the media and from some professional commentators, we found that lower carbohydrate intake was associated with significantly greater odds of T2DM and higher HbA1c concentration. Higher adherence to conventional dietary recommendations (basing meals on starchy carbohydrates, lower fat and saturated fat contents, eating more fruits and vegetables) was associated with lower HbA1c concentration.

Few longitudinal cohort studies have evaluated the links between LCHF diets and long-term risk of T2DM, with inconclusive findings [15, 16, 27]. The LCHF dietary pattern was associated with higher T2DM risk in men, women with history of gestational diabetes [15, 27], but another found no association in women without gestational diabetes [16]. A meta-analysis of four prospective studies with follow-up durations 3.6 to 20 years [28] showed no evidence of association between LCHF (highest quantile of LCHF score) and T2DM risk (pooled RR 1.17; 95% CI 0.90–1.51) but this small study had high heterogeneity (*I*^2^ = 81.5%, *P* < 0.001). The authors did a subgroup analysis in those cohort studies with adjustment for total energy intake (*n* = 3 of 4) and found improvement in heterogeneity (pooled RR 1.32; 95% CI 1.05–1.75; *I*^2^ = 0%) [28]. All included studies (*n* = 4) were assessed as Newcastle–Ottawa Scale ≥5 for methodological quality, but a subgroup analysis by study quality (score > 7 and ≤7) revealed an association only in lower-quality studies (score ≤7; *n* = 2/4; pooled RR 1.31; 95% CI 1.15–1.50; *I*^2^ = 0%), with no association in two higher quality studies (score > 7; pooled RR 1.09; 95% CI: 0.73–1.63; *I*^2^ = 86%) [28]. The evidence on this topic is clearly scarce and varies in quality, and dietary assessment quality is a limiting factor. All prospective studies used food frequency questionnaires for dietary assessment, which are less reliable than the 4-day food diary used in the UK NDNS survey. While cross sectional in nature, our study and indings link LCHF diets to both T2DM and HbA1c level, and therefore add to the findings of previous cohort studies.

A beneficial relationship between adherence to dietary recommendations and lower HbA1c and T2DM risk is reassuring, and in keeping with previous literature. Another recent UK cross-sectional study showed similarly that adherence to UK dietary guidelines was associated with lower HbA1c and T2DM risk [23]. Also, findings from prospective studies showed that adherence to dietary guidelines, increased fruits and vegetables and whole grain intakes were associated with 7–30% lower risk of T2DM [29–32]. Moreover, a secondary analysis of the Women’s Health Initiative Dietary Modification Trial showed that low-fat diet, with increased grains, fruits and vegetables, reduced the rate of conversion from normoglycemia to impaired fasting glucose by 25% after 8-year intervention [4]. Notably, participants in this trial increased carbohydrate intake by ~ 8% of energy, with a corresponding reciprocal decrease in fat intake of ~ 8%. This also supports the assertion that greater carbohydrate intake, particularly from grains and fruits, could delay progression to T2DM [4].

Other mechanisms (e.g., oxidative stress, peripheral insulin resistance), aside from carbohydrates intake and postprandial glycemia, may contribute to elevation of HbA1c and might explain the association with lower carbohydrate diets. Avoiding carbohydrate foods like whole grains, fruits and some starchy vegetables inevitably lowers micronutrients and bioactive compounds, fiber, and some minerals (e.g., magnesium) [14]. Lower fruits and associated nutrients and bioactives, alongside higher fat intake, could impact on oxidative stress [33–37]. High oxidative stress consequently enhances protein glycation, including the formation of fructosamine and HbA1c. Vlassopoulos et al. modelled that under normal glucose concentration, higher oxidative stress increases fructosamine production by 35%, compared to non-oxidative state [38].

Oxidative stress, as well low magnesium status and high fatty acids concentration (from high-fat intake), also raises insulin resistance through an alteration in the insulin receptor signaling pathway [39, 40]. A recent well-controlled crossover isocaloric feeding study in adults with overweight/obesity reported a decline in insulin sensitivity, reduction in glucose disposal rate by − 0.37 ± 0.15 mmol/min measured by hyperinsulinemic-euglycemic clamp, after 4 weeks of high-fat/high-saturated fat diet compared to a low-fat diet (glucose disposal rate + 0.12 ± 0.11 mmol/min, *P* < 0.01) [41]. All factors discussed above together with chronic inflammatory state in obesity could explain the associations between LCHF diet, high saturated fat intake and high HbA1c that were robust in a subgroup of individuals with BMI ≥ 25 kg/m^2^. Consistently, recent meta-analyses have shown that low magnesium intake was associated with higher risk of T2DM by 17–22% [42, 43].

This study has limitations that must be considered when evaluating its findings. This study could not refer to very low-carbohydrate ketogenic diets, as there are no data on ketosis status for this dataset. Notably, there is no standardized definition of LCHF diets, although < 26%E CHO has been proposed as a cut-off value [44]. Only 0.24% of the study sample (*n* = 8/3234) consumed carbohydrates below this threshold, a finding comparable to data from the UK Biobank (0.34% of participants) [45]. As with all cross-sectional studies, residual confounders and reverse causation could not be excluded; for example, the association between low-carbohydrate intake and T2DM prevalence could be explained by that those with diabetes might have changed their dietary behaviour by reducing carbohydrate intake. We, therefore, conducted analyses in a population mainly without diabetes, including in-depth sensitivity analyses and used a biomarker (HbA1c) as an outcome to minimize bias. The LCHF score is arbitrary. However, this score allows a practical approach to assess dietary pattern, reflecting the complementarity of food/nutrient intakes rather than focusing on single nutrients that are never consumed in isolation [46]. Finally, we could not distinguish between type 1 and type 2 diabetes, including those with insulin use, in the present study. However, the prevalence of type 1 diabetes is approximately 0.6% of UK population (about 10% of people with diabetes in the UK [26]); any individuals with type 1 diabetes in the sample could have little impact on our analyses, as shown through exclusion simulations in our sensitivity analyses.

A key strength of our study is its use of the UK nationally representative data for hypothesis testing on a topic of substantial scientific and public interest. Results could, therefore, be generalizable to the UK population and possibly to other countries, as dietary recommendations (to eat more whole grain, fruits and vegetables, and lower fat, saturated fat, sodium and sugar) are rather similar across many countries [47, 48]. Using prospective dietary records does not rely on memory so may provide more accurate estimates of actual intakes than food frequency questionnaires.

Regarding diabetes prevention, our cross-sectional results oppose the simplistic notion that lower carbohydrate contents in the diet per se could lower HbA1c concentration, particularly in people without diagnosed diabetes. Long-term, high-quality randomized controlled trials of LCHF diets on diabetes prevention are needed before such diets should be recommended.

## Electronic supplementary material

Below is the link to the electronic supplementary material.

Supplementary material 1 (DOCX 243 kb)

## Abbreviations

T2DM: Type 2 diabetes
LCHF: Low-carbohydrate, high-fat diet
NDNS: National diet and nutrition survey
DRV: Dietary reference value
NMES: Non-milk extrinsic sugar
NSP: Non-starch polysaccharide
BMI: Body mass index
NS-SEC: National statistics socio-economic classification
