# Supplementary material for: Lower carbohydrate and higher fat intakes are associated with higher hemoglobin A1c: findings from the UK National Diet and Nutrition Survey 2008–2016
Source: Eur J Nutr. 2019 Nov 4;59(6):2771–82. doi: 10.1007/s00394-019-02122-1 (PMC7413867; doi:10.1007/s00394-019-02122-1)
Supplement: Supplementary file 1 — Supplementary material 1 (DOCX 243 kb) [file 394_2019_2122_MOESM1_ESM.docx]

**Lower carbohydrate and higher fat intakes are associated with higher hemoglobin A1c: findings from the UK National Diet and Nutrition Survey 2008-2016.**

European Journal of Nutrition

Chaitong Churuangsuk ^1^, Michael E.J. Lean ^1^ , Emilie Combet ^1 *^

^1^ Human Nutrition, School of Medicine, Dentistry and Nursing, College of Medical, Veterinary and Life Sciences, University of Glasgow, New Lister Building, Glasgow Royal Infirmary, 10-16 Alexandra Parade, Glasgow, G31 2ER, UK

**Corresponding author ***:

Dr Emilie Combet, Room 2.22, Level 2, New Lister Building, 10-16, Alexandra Parade, Glasgow Royal Infirmary, Glasgow, G31 2ER.

E-mail: [Emilie.combetaspray@glasgow.ac.uk](mailto:Emilie.combetaspray@glasgow.ac.uk)

**Online Resource Table 1** Scoring systems for adherence to low-carbohydrate diet and UK recommendations.

| **Point** | **CHO % food E** | **Fat %food E** |
| --- | --- | --- |
| 10 | ≤39.49 | >42.91 |
| 9 | ≤42.23 | ≤42.91 |
| 8 | ≤44.31 | ≤40.35 |
| 7 | ≤46.00 | ≤38.60 |
| 6 | ≤47.49 | ≤37.11 |
| 5 | ≤48.74 | ≤35.63 |
| 4 | ≤50.12 | ≤34.33 |
| 3 | ≤51.80 | ≤33.00 |
| 2 | ≤53.65 | ≤31.48 |
| 1 | ≤56.37 | ≤29.56 |
| 0 | >56.37 | ≤26.63 |

Each category contains equal number of participants

**Online Resource Table 2** Scoring systems for adherence to UK recommendations.

| **Point** | **Carbohydrate (%E)** | **Non-starch polysaccharides (g/d)** | **Fruits & Vegetables (g/d)** | **Fish (g/d)** | **Sodium (mg/day)** | **Fat (%E)** | **Saturated Fat (%E)** | **Non-milk extrinsic sugar (%E)** |
| --- | --- | --- | --- | --- | --- | --- | --- | --- |
| 0 | ≤47.50 | ≤17.10 | ≤380 | ≤38 | >2520 | >36.75 | >11.55 | >11.55 |
| 1 | ≤52.50 | ≤18.90 | ≤420 | ≤42 | ≤2520 | ≤36.75 | ≤11.55 | ≤11.55 |
| 2 | >52.50 | >18.90 | >420 | >42 | ≤2280 | ≤33.25 | ≤10.45 | ≤10.45 |

**Online Resource Table 3** Prevalence of type 1 diabetes mellitus (in all patients with diabetes) stratified by age groups (National Diabetes Audit Report 2017-2018).

| **Age groups (years)** | **Prevalence of T1DM in all patients with diabetes (%)** | **Numbers of participants with diabetes in the NDNS dataset**  **(n=104)** | **Numbers of participants with diabetes to be excluded.**  **(n=10)** |
| --- | --- | --- | --- |
| **20-29** | 67.4 | 6 | 4 |
| **30-39** | 29.3 | 5 | 1 |
| **40-49** | 12.7 | 14 | 2 |
| **50-59** | 6.7 | 25 | 2 |
| **60-69** | 3.4 | 29 | 1 |
| **≥70** | - | 25 | - |

**Online Resource Table 4:** Sensitivity analysis for Odds Ratio of T2DM.

|  | **Main analysis**  (n=3234) | | |  | **Sensitivity analysis 1**  excluding participants with diabetes, aged ≤30 years  (n=3228) | | |  | **Sensitivity analysis 2**  running the logistic regression model 1000 times, each time randomly excluding 10% of participants with diabetes for possible T1DM  (n=3224) | |
| --- | --- | --- | --- | --- | --- | --- | --- | --- | --- | --- |
| **Exposures** | **Odds Ratio^1^** | **95%CI** | ***P*** |  | **Odds Ratio^1^** | **95%CI** | ***P*** |  | **Mean**  **Odds Ratio^1^** | **95%CI ^4^** |
| *Macronutrients ^2^* |  |  |  |  |  |  |  |  |  |  |
| **Carbohydrate** | 0.88 | 0.78 - 0.99 | 0.030 |  | 0.88 | 0.78 – 0.99 | 0.031 |  | 0.8782 | 0.878 – 0.879 |
| **Fat** | 1.17 | 1.02 - 1.33 | 0.022 |  | 1.17 | 1.03 – 1.34 | 0.019 |  | 1.1720 | 1.171 – 1.173 |
| **Saturated fat** | 1.14 | 0.90 - 1.46 | 0.270 |  | 1.15 | 0.90 – 1.47 | 0.263 |  | 1.1536 | 1.152 – 1.155 |
| **Protein** | 1.01 | 0.81 - 1.26 | 0.920 |  | 1.00 | 0.80 – 1.26 | 0.999 |  | 0.9974 | 0.996 – 0.999 |
| *Adherence score ^3^* |  |  |  |  |  |  |  |  |  |  |
| **LCHF score** | 1.08 | 1.02 - 1.14 | 0.006 |  | 1.08 | 1.02 – 1.14 | 0.005 |  | 1.0801 | 1.0797 – 1.0804 |
| **DRV score** | 0.95 | 0.85 - 1.06 | 0.350 |  | 0.95 | 0.85 – 1.06 | 0.354 |  | 0.9481 | 0.947 – 0.949 |

**^1^** adjusted for age, sex, BMI, ethnicity, smoking status, socioeconomic status, survey years, total energy intake.

**^2^** per 5% food energy increment.

**^3^** per 2-point score increment.

^4^ 95%CI of the mean Odds Ratio

**Online Resource Table 4:** Sensitivity analysis for Odds Ratio of T2DM. ***(Continued)***

|  | **Main analysis**  (n=3234) | | |  | **Sensitivity analysis 3**  Unknown T2DM prevalence after excluding individual with known T2DM  (n=3130) | | |  | **Sensitivity analysis 4**  T2DM prevalence after excluding individual with T2DM who were on weight loss diet  (n=3198) | | |
| --- | --- | --- | --- | --- | --- | --- | --- | --- | --- | --- | --- |
| **Exposures** | **Odds Ratio^1^** | **95%CI** | ***P*** |  | **Odds Ratio^1^** | **95%CI** | ***P*** |  | **Odds Ratio^1^** | **95%CI** | ***P*** |
| *Macronutrients ^2^* |  |  |  |  |  |  |  |  |  |  |  |
| **Carbohydrate** | 0.88 | 0.78 - 0.99 | 0.030 |  | 0.93 | 0.79 - 1.09 | 0.374 |  | 0.87 | 0.76 – 0.99 | 0.032 |
| **Fat** | 1.17 | 1.02 - 1.33 | 0.022 |  | 1.15 | 0.95 - 1.38 | 0.142 |  | 1.19 | 1.04 – 1.38 | 0.015 |
| **Saturated fat** | 1.14 | 0.90 - 1.46 | 0.270 |  | 1.16 | 0.82 - 1.62 | 0.405 |  | 1.19 | 0.92 – 1.54 | 0.175 |
| **Protein** | 1.01 | 0.81 - 1.26 | 0.920 |  | 0.86 | 0.62 - 1.20 | 0.378 |  | 0.97 | 0.76 – 1.24 | 0.809 |
| *Adherence score ^3^* |  |  |  |  |  |  |  |  |  |  |  |
| **LCHF score** | 1.08 | 1.02 – 1.14 | 0.006 |  | 1.06 | 0.98 - 1.14 | 0.155 |  | 1.08 | 1.02 – 1.14 | 0.010 |
| **DRV score** | 0.95 | 0.85 - 1.06 | 0.350 |  | 0.99 | 0.84 - 1.15 | 0.858 |  | 0.91 | 0.80 – 1.02 | 0.111 |

**^1^** adjusted for age, sex, BMI, ethnicity, smoking status, socioeconomic status, survey years, total energy intake.

**^2^** per 5% food energy increment.

**^3^** per 2-point score increment.

^4^ 95%CI of the mean Odds Ratio

**Online Resource Table 5** Subgroup analysis in non-diagnosed diabetes participants (n=3130) with BMI <25, ≥25 kg/m^2^ showing associations between %HbA1c level and macronutrients and diet scores.

|  | **HbA1c ≥5.5%** |  |  |  | **%HbA1c concentration** |  |  |  |  |
| --- | --- | --- | --- | --- | --- | --- | --- | --- | --- |
| **Predictors** | **Odds Ratio^1^** | **95%CI** | **p-value** |  | **Β^1^** | **SE** | **Lower 95% CI** | **Upper 95% CI** | **p-value** |
| **BMI < 25 kg/m^2^ (n=1258)** |  |  |  |  |  |  |  |  |  |
| *Macronutrients ^2^* |  |  |  |  |  |  |  |  |  |
| **Carbohydrate** | 0.95 | 0.85 - 1.05 | 0.30 |  | -0.011 | 0.009 | -0.028 | 0.006 | 0.200 |
| **Fat** | 1.10 | 0.98 – 1.23 | 0.11 |  | 0.018 | 0.009 | 0.000 | 0.037 | 0.053 |
| **Saturated fat** | 1.19 | 0.97 – 1.46 | 0.09 |  | 0.039 | 0.017 | 0.006 | 0.072 | ***0.021*** |
| **Protein** | 0.92 | 0.75 – 1.13 | 0.43 |  | -0.015 | 0.016 | -0.047 | 0.016 | 0.341 |
| *Adherence score ^3^* |  |  |  |  |  |  |  |  |  |
| **LCHF score** | 1.02 | 0.98 – 1.07 | 0.37 |  | 0.006 | 0.004 | -0.002 | 0.013 | 0.122 |
| **DRV score** | 0.95 | 0.87 – 1.04 | 0.25 |  | -0.010 | 0.008 | -0.025 | 0.004 | 0.164 |
|  |  |  |  |  |  |  |  |  |  |
| **BMI ≥ 25 kg/m^2^ (n=1872)** |  |  |  |  |  |  |  |  |  |
| *Macronutrients ^2^* |  |  |  |  |  |  |  |  |  |
| **Carbohydrate** | 0.91 | 0.84 – 0.99 | ***0.024*** |  | -0.020 | 0.009 | -0.038 | -0.002 | ***0.031*** |
| **Fat** | 1.18 | 1.08 – 1.29 | ***<0.001*** |  | 0.035 | 0.010 | 0.015 | 0.054 | ***0.001*** |
| **Saturated fat** | 1.37 | 1.16 – 1.63 | ***<0.001*** |  | 0.052 | 0.019 | 0.014 | 0.089 | ***0.007*** |
| **Protein** | 0.87 | 0.75 – 1.00 | 0.053 |  | -0.029 | 0.016 | -0.061 | 0.004 | 0.081 |
| *Adherence score ^3^* |  |  |  |  |  |  |  |  |  |
| **LCHF score** | 1.06 | 1.02 – 1.09 | ***0.003*** |  | 0.012 | 0.004 | 0.004 | 0.020 | ***0.003*** |
| **DRV score** | 0.87 | 0.81 – 0.94 | ***<0.001*** |  | -0.027 | 0.008 | -0.043 | -0.011 | ***0.001*** |

**^1^** adjusted for age, sex, BMI, ethnicity, smoking status, socioeconomic status, survey years, total energy intake.

**^2^** per 5% food energy increment.

**^3^** per 2-point score increment.


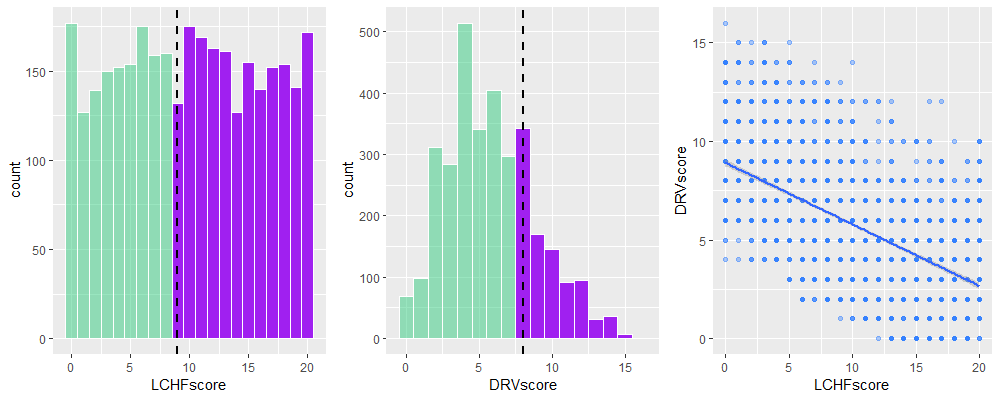


**Online Resource Figure 1. Distributions of low-carbohydrate high fat score (LCHF) and dietary reference values score (DRV) and their correlation.**

*Colored area divides subjects who met recommendations: LCHF score of 9 and above reflects <50%food energy from carbohydrate and >35%food energy from fat; and DRV score of 8 and above reflects meeting each item of food and nutrient intakes in the UK recommendations.*

**^
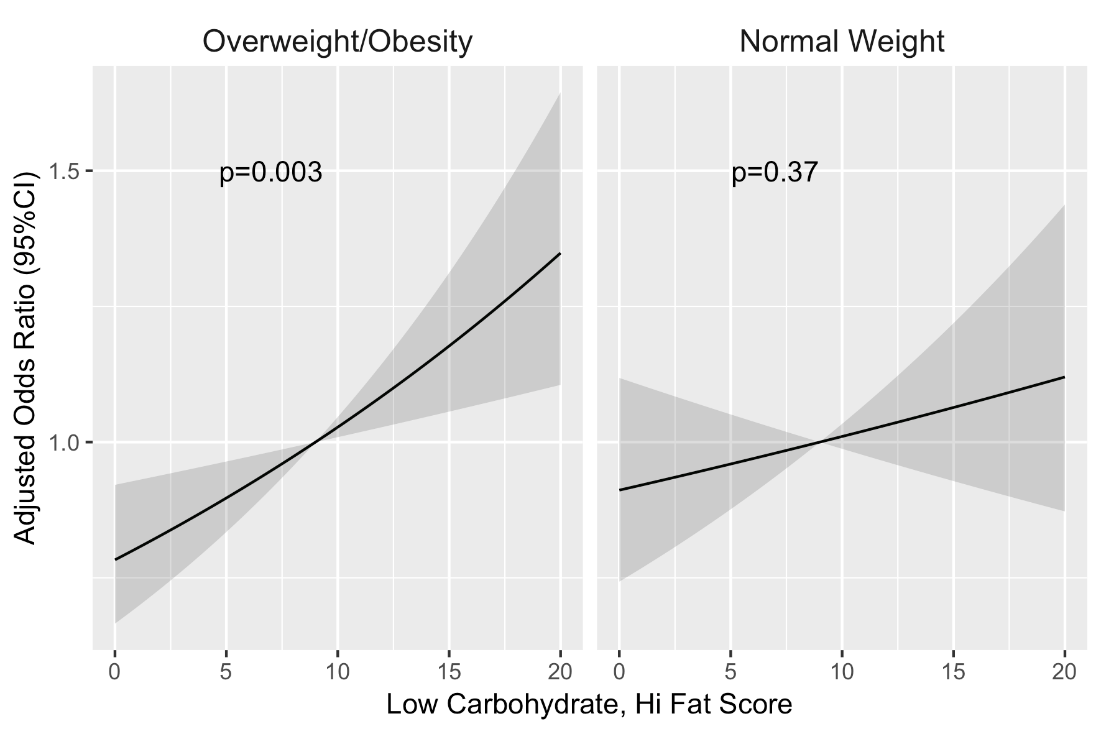

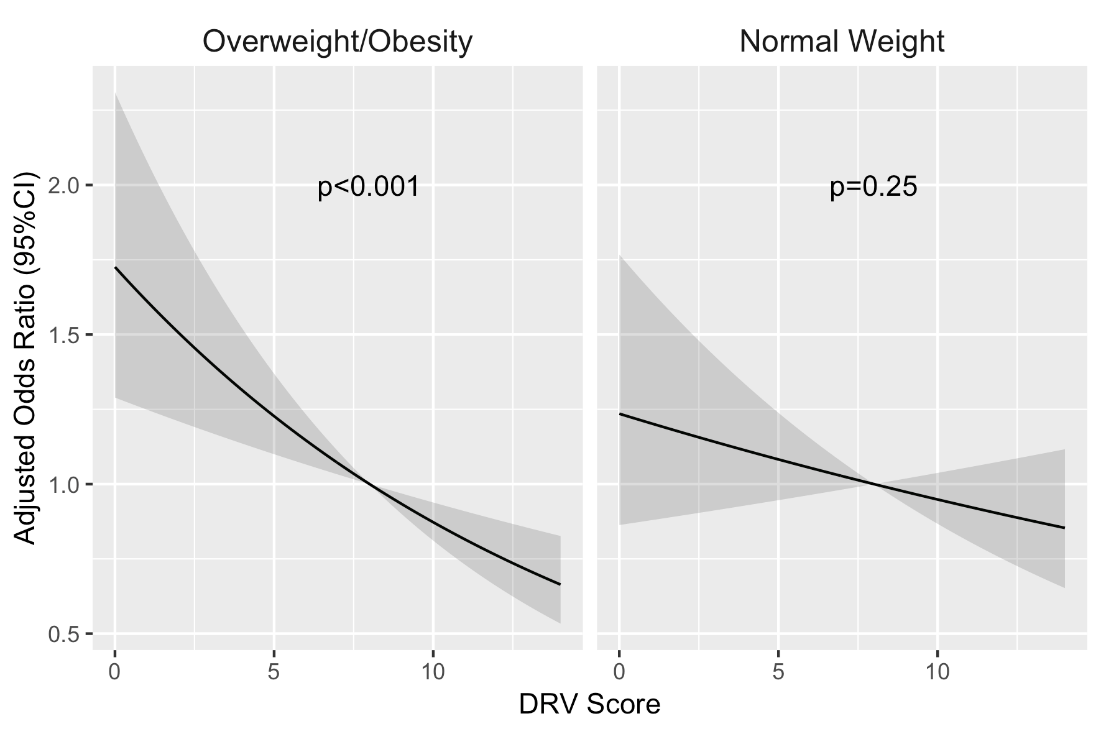
^**

**Online Resource Figure 2** Odds ratio of elevated HbA1c ≥5.5% by subgroup analysis in participants without diagnosed diabetes (n=3130) with BMI<25 kg/m^2^ [n=1258], BMI≥ 25 kg/m^2^ [n=1872]. Adjustment for age, sex, BMI, Ethnicity, smoking status, NS-SEC, total energy intake and survey years
